# Towards Praxes of the Region: Agential Constructivist Approaches to Regionalisms

**DOI:** 10.1177/00207020231152250

**Published:** 2023-01-29

**Authors:** J. Andrew Grant

**Affiliations:** Department of Political Studies, 210177 Queen’s University, Kingston, ON, Canada

**Keywords:** Africa, regionalism, violent conflict, natural resources, regional organizations, norms, theory and practice, informal economies, agency, constructivism

## Abstract

While both formal and informal regionalisms examine the political ramifications of economic flows of capital, goods, and people, there is a blurring of such conceptual dichotomies in practice. Hence, in order to offer a more accurate account of the distinctions and overlap between the formal and informal – and to rectify the tendency to overlook the agency of African state actors and non-state actors – this article offers an agential constructivist approach that seeks to advance a praxis – or praxes – of the region. To that end, the article advances the concept of bifurcated interregionalisms as a means of analyzing cases of regional dynamics and regionalisms in Southern Africa and East Africa. The article concludes by offering some reflections on the future of regionalisms in an emerging global order in flux whereby illiberal and xenophobic variants of regionalisms compete with the liberal and cosmopolitan versions.

In recent years, scholars and practitioners have devoted greater attention to regions as vital agents of economic development. Regions can be quite modest in size—micro-regions as small as municipalities or macro-regions as large as West Africa exhibit compelling political economies of global development and human security. In the case of the former, the role and regional implications of cities as engines of economic growth have attracted much scholarly attention over the past decade. What is more, regional development corridors covering amorphous geographical regions are yet another intriguing form of regionalisms. These dynamics and the governance challenges and opportunities such regions generate fall under the aegis of what may be referred to as regionalism studies, which is often categorized as either formal (state-led, state-centric, policy-driven) or informal (state *and* non-state actors as drivers, poly-centric, challenges policy) in terms of focus. While both formal and informal regionalism—more accurately conceptualized in the plural as regionalism***s*—**examine the political ramifications of economic flows of capital, goods, and people, there is a blurring of such conceptual dichotomies in practice. Hence, in order to offer a more accurate account of the distinctions and overlaps between the formal and informal—and to rectify the tendency to overlook the agency (or agencies) of African state and non-state actors—this article offers an agential constructivist approach that seeks to advance a praxis—or praxes—of the region. Next, the empirical core of the article, which is based in part on primary sources such as fieldwork including participant observations,^1^ provides some analyses of the regional dynamics and regionalisms drawn from different parts of Africa. Specifically, the concept of bifurcated interregionalisms is introduced and applied to two evocative, multi-layered case studies: the Mozambican province of Cabo Delgado and Ethiopia’s Tigray region, and their respective regional environments. The article concludes by offering some reflections on the future of regionalisms in an emerging global order in flux with the forces of “new nationalism” generating an illiberal and xenophobic variant of regionalisms that run counter to the liberal and cosmopolitan type that “global governors” and “global actors” sought to promote at global, regional, and local levels.

## Epistemological and ontological connective “sinews and tissues” within the study of regionalisms

It is tempting for social scientists to analyze the causes, effects, and outcomes associated with a particular puzzle or issue-area, and then offer generalizations and comprehensive conclusions about that particular phenomenon. Even the most reflexive social scientist might succumb to this temptation because it provides a reassuring degree of problem-solving and knowledge creation. Scholars of regionalism are no different; pronouncements of findings regarding a particular form of regionalism abound. However, such understandings of regionalism offer a somewhat myopic view of the complexity and many “sides” of regionalism. Timothy Shaw has encouraged scores of scholars to pursue epistemological and ontological pluralism in their work.^2^ Arguably, his best-known pluralization is regionalism*s*, which he invokes as a means of reminding scholars to consider key approaches, categories, and concepts in the plural. Indeed, in order to recognize the epistemological, ontological, and practical/on-the-ground diversity, differences, and positionalities that inform our analyses, it is preferable to consider and speak of globalizations, femininities, masculinities, Global Norths, Global Souths, Africas, COVID-19s, and the like. Consider, for example, how female executives working for transnational corporations in Luanda experience COVID-19 differently than their counterparts managing confectionaries on the ground floor of those office buildings. With these diversities, differences, and positionalities in mind via a reflexive pluralist approach—which shares some of the pragmatic pluralism advanced by W.R. Nadège Compaoré et al.^3^—the article now proceeds to offer some definitions of key terms and concepts. The discussions in the following sub-sections will illustrate some of the debates and terminologies—not to mention the epistemological and ontological connective “sinews and tissues”—within the study of regionalisms.

### Praxis and agential constructivism (or praxes and agential constructivisms)

I conceptualize praxis—or praxes in the plural—in a manner that is influenced by the work of Hannah Arendt and her approach to praxis insofar as it emphasizes the action^4^—or agency, in my view—of actors who comprise social constructs, such as regions in both their informal and formal manifestations. Praxes also complement the discussion on theory and practice that Larry Swatuk advances.^5^ As he notes, actors—including practitioners—may be unaware of the relationship between theory and action, and hence unaware of the praxes informing their strategies and decision-making as they carry out their responsibilities.^6^ In other words, there are theories of regionalisms, but most of the post-1990s scholarship emphasizes the agency of such phenomena with comparatively less focus on the structures of regions and regionalisms. This is not to say that the structural elements of regionalisms have been discarded. Rather, there has been greater operationalizing of the concept of regionalisms via studies of agency of the actors involved in extant and evolving processes. Praxes of regions and regionalisms are also consistent with the concept of “regionness” advanced by the scholarship of Björn Hettne and Fred Söderbaum^7^ more than two decades ago. That is, regionness connotes “the process whereby a geographical area is transformed from a passive object to an active subject capable of articulating the transnational interests of the emerging region.”^8^ Reference to the process—or processes—of regionness is key because it draws attention to the constituent actors and “implies that a region can be a region ‘more or less’” and that we can conceptualize how the “level[s] of regionness can both increase and decrease.”^9^ Concomitantly, regionness draws attention to the agency of constituent actors.

The above conceptualization of praxes also aligns well with agential constructivism—or agential constructivism*s****—***given their mutual emphases on theorizing the agency of actors. Agential constructivism seeks to refine the IR scholarship on constructivism of the past two-plus decades by drawing greater attention to the agency of actors. Importantly, agential constructivism is *not* calling on scholars to ignore structure; rather, by encouraging scholars to reflect on practical considerations, to consider “on the ground” ramifications of global forces, and to assess the “everyday” experiences and practices of actors,^10^ the aim is to provide a more complete understanding of social scientific phenomena—for example, regionalization and regionalisms in the context of the present study. One of the distinctive traits of agential constructivisms is that they provide greater “optimism” about the emancipatory potential of actors to possibly overcome—or at least make positive headway towards overcoming—structural constraints as well as to provide voices to those groups who have been silenced or to empower those traditionally marginalized groups who exude a kind of power and influence by choosing to be purposely silent at key governance junctures, the latter of which is highlighted in the work of Erin Baines^11^ and Jane Parpart.^12^ Another distinctive element is that they open up greater “epistemological space” for scholars to delve into femininities, masculinities, Global Norths, and Global Souths—via rich case studies or in comparative context—which “traditional” constructivism—at least as promulgated by Alexander Wendt^13^ and Nicholas Onuf,^14^ has largely lacked (and is only recently being “rectified” by those seeking to refine the work of these two leading constructivist scholars). It should also be noted that agential constructivisms retain “traditional” constructivism’s study of norms, conventions, practices, symbols, and language as well as some of the latter’s variants that do not discard the importance of material considerations. Put differently, agential constructivisms acknowledge that the material considerations that underpin global political economies, global governance, and power differentials among actors certainly inform socially constructed considerations—dialectally from the local to the global—and vice versa.^15^ This further reinforces agential constructivisms’ concern for how actors combine theory and practice, even if unknowingly, as part of their expectations about how daily events will unfold on the ground.

### Regions, regionalisms, and bifurcated interregionalisms

Timothy Shaw’s scholarly work on regionalisms has spanned more than two decades.^16^ His solo-authored and collaborative works have had a significant influence on scholars in the subfield.^17^ His work informs the “connective sinews and tissues” that link regions and regionalisms,^18^ as well as more recent theoretical and conceptual innovations such as bifurcated interregionalisms (as defined below).^19^ For Shaw and other “new regionalists,” the region represents more than mere geographical and governmental/administrative demarcations. Rather, it goes beyond such conceptions in order to emphasize the socially constructed scope within which residents engage in socioeconomic activities, ranging from the licit to the illicit and the peaceful to the violent—with much variance between these extremes. This conception of the region and its oft-amorphous shape recognizes the agency of its constituent actors, which is further operationalized when invoking the term “regionalisms.” What is more, regionalisms often have cross-cutting and overlapping scopes and breadths, with varying degrees of intensity, which, like their constituent actors, can fluctuate from day to day, month to month, year to year, and decade to decade.

As noted above, one of the more recent innovations in the study of regionalisms is the identification of bifurcated interregionalism.^20^ Moving beyond formal interregionalism^21^ and drawing upon quasi-interregionalism^22^ and stealth interregionalism,^23^ agency figures prominently in the study of bifurcated interregionalism—or bifurcated interregionalism*s*—as it seeks to reveal and explain the *interactions and contestations between* “agents of formal regionalism and informal regionalism.”^24^ Although this definition of the concept of bifurcated interregionalisms has been applied to African cases—underscoring the continent’s importance for scholars seeking to understand order in the international system—it is also important insofar as its conceptual robustness could be readily applied to a wide variety of other cases within and beyond Africa. This article now applies such theorizing to empirical analyses of contemporary cases of violent campaigns in Southern Africa and East Africa. These cases are neither representative samples in the quantitative sense of the term, nor able to satisfy the historian’s desire to account for myriad historical details. Rather, the aim is to provide evocative cases that are instructive for scholars and observers of bifurcated interregionalisms.

## Bifurcated interregionalisms in Southern Africa and East Africa

Southern Africa and East Africa are home to several “simmering” conflicts, defined as those protracted conflicts that are characterized by episodic spikes of violence that interrupt extended periods of relative calm and create gnawing insecurity for civilian populations. Within Southern Africa and East Africa, respectively, conflicts in the Cabo Delgado province of Mozambique and the Tigray Region of Ethiopia and their environs have sparked much discussion about their root causes, responses, and regional dynamics involving both state and non-state actors. While the two conflicts are anchored in different histories, colonial experiences, regional hegemons, and regional intergovernmental organizations, they have common types of internal and external influences in terms of formal and informal regional dynamics, bifurcated interregionalisms, and the potential responses to tackle human security considerations. Although it is tempting to describe the regional dynamics in these two cases as complex, a more accurate assessment would be that other past and current conflicts have merely exhibited regionalisms that were downplayed by observers and scholars alike. Nevertheless, unpacking informal and formal dimensions will help demonstrate that the conflicts are driven largely by socioeconomic factors.

### Cabo Delgado

Since circa October 2017, the Mozambican province of Cabo Delgado, located in the northeastern-most part of the country, has been home to a violent insurgency that has brought in or involved state and non-state actors from within and beyond the continent. On the one hand, informal regional relations have manifested through the insurgency. Jessica Trisko Darden and Emily Estelle, for instance, demonstrate that external dynamics have shaped the local, separatist Salafi movement that emerged in northeast Mozambique from the start of the conflict.^25^ Initially established as a peaceful movement, Ansar al-Sunna rapidly evolved into a non-state armed group (NSAG) that laid the groundwork for future militarization in the region. The influence of external forces through the recruitment of fighters to this Islamic State (ISIS)-affiliated insurgent group underscores the regionness of the conflict. The general consensus among observers is that Ansar al-Sunna has linkages with other NSAGs based in Somalia, Kenya, Uganda, Tanzania, Sudan, the Democratic Republic of Congo (DRC), and Saudi Arabia.^26^ Many of these combatants are reported to have studied, trained, or obtained literature from one or more NSAGs or other non-state group based in these countries.^27^ There are reports of NSAG camps operating in northern Tanzania, the African Great Lakes Region (GLR), Kenya, and Somalia—where young recruits are given religious and military training. What is more, a recent UN Security Council report suggested that the Islamic State’s branch in northern Somalia serves as the command centre for the branches in the DRC and Mozambique.^28^ In contrast to “networks for peace,”^29^ these “networks of terror” combine ideological, religious, and military identity-building forces to create a form of regionness in this corner of Southern Africa. This reinforces agential constructivism’s concern with the influence of ideas, symbols, and practices—which are operationalized in networks of terror. Like other NSAGs and their networks located in other parts of the globe, some degree of self-financing is required to support the network in question. Illicit trade in timber, charcoal, ivory, and rubber—involving state and non-state partners in Tanzania, the GLR, China, and Vietnam^30^—provides important financial sustenance. These “networks of plunder”^31^ are vital economic vectors (material considerations) that have the potential to take precedence over the social vectors (e.g., ideological and religious motivations) of the constituent NSAGs—an interplay of considerations accounted for by agential constructivism (as defined earlier in the article).

On the other hand, formal regional relations have also ramped up in response to the violent conflict in Cabo Delgado and the surrounding region. Delegates from neighbouring states have sought to formulate a regional counterterrorism strategy. At the Southern African Development Community’s (SADC) Extraordinary Organ Troika Summit, these delegates agreed to provide support to the Mozambique government to help curb the rising volatility and militarization in the region.^32^ Although initially the formal regional response to the conflict was in the form of providing humanitarian assistance, the heightened situation has led several countries—namely Angola, Botswana, member-states of the European Union, Rwanda, South Africa, the United States, and others—to deploy soldiers, equipment, and military advisers to help fight the NSAG insurgents.^33^

Although the influx of the human and material resources as agents of formal regionalism (e.g., the SADC, the EU) has decreased the degree of violence in the region, the interactions and contestations with the NSAGs supported by informal regionalisms mean that bifurcated interregionalisms persist in and around Cabo Delgado. Illiberal regionness with illiberal regional objectives persists—a relatively recent trend that mirrors the influence of Salafi-jihadi insurgencies such as the one in Mozambique’s Cabo Delgado province, but also in Egypt, Nigeria (by groups largely affiliated with al-Qaeda and the Islamic State), and other parts of Africa. The drivers of bifurcated interregionalisms in these countries have raised major security and development concerns.^34^ In fact, in Cabo Delgado, which has long been home to significant volumes of oil deposits, liquefied natural gas fields, and gemstones, there have been disruptions in the operations of large transnational oil firms—namely, Total and Exxon Mobil. Undoubtedly, these non-state actors will continue to press state actors, informally and behind the scenes, to improve the regional security environment in and around Cabo Delgado. Although this region is rich in natural resources that can significantly contribute to community development and national development more broadly,^35^ a clear-eyed assessment of the near-future prospects for regional security is that transnational natural resource sector firms are not necessarily interested in embracing equitable outcomes for local communities as a primary objective. For agential constructivists, the constituent norms of corporate social responsibility have become more important for natural resource sector firms in recent decades—but these scholars concede that there are limits on the emancipatory potential of operationalizing such norms in terms of elevating equitable outcomes of local communities above substantial profit. Furthermore, such firms may rely increasingly upon private security companies (either in-house or externally sourced), which generates symbolic and material ramifications of concern to agential constructivists as well as governance challenges in terms of human security and development considerations for local communities.^36^

### Tigray

Since November 2020, the Ethiopian region of Tigray, located in the northernmost part of the country, has been the epicentre of a particularly vicious civil war. Exhibiting the dangers of ethno-federalism, the civil war has a regional dimension (in the form of Eritrean participation, among other state and non-actors) as well as more conventional socioeconomic dynamics.^37^ As Dedefo Bedaso^38^ and others^39^ point out, Ethiopia is a place of diverse cultures, languages, and ethnicities. Within this context, the exclusion of particular ethnic groups from power serves as a breeding ground for violent-conflict-inducing grievances. Bedaso expands on this premise by attributing the onset of the conflict to the fact that “diverse ethnicities have been forcefully marginalized, subjugated, and assimilated to accept the system that does not represent them.”^40^ Although ethno-federalism may be portrayed as a way to promote self-determination and self-autonomy—all infused by liberal norms—it is by no means a guarantee against the type of illiberal outcomes we are witnessing in and around the Tigray region of Ethiopia.

The socioeconomic elements of the conflict in Tigray are not as explicit as in Cabo Delgado, where there is anticipated development of hydrocarbon and mineral reserves, but this does not mean they are absent. Rather, the global political economies of the following developments have regional and domestic considerations. For instance, the Grand Ethiopian Renaissance Dam will have myriad socioeconomic repercussions, ranging from hydropower exports to possibly constraining agricultural, food security, and other water-usage considerations for Sudan and Egypt within the regional environs of the Nile River Basin. Regional security concerns stemming from tense Sudan-Ethiopia relations over the former accepting fleeing Tigrayans—with numbers exceeding 60,000 and expected to grow—exacerbate an already fraught relationship between the two countries owing to disputes concerning the bordering land in the al-Fashaga region.^41^ Domestically, the socioeconomic considerations are linked to the benefits of patronage by holding key posts in the government, civil service, and other sectors of the economy. By forcibly removing Tigrayans from such positions and violently repressing them, the Abiy Ahmed regime is taking advantage of such tumultuous conditions to insert those perceived to be more favourable to patron-client networks that benefit the regime. From the perspective of the Abiy Ahmed regime, this is also a form of payback based on a perception that Tigrayans had previously benefitted from such patron-client networks in terms of greater access to public goods—such as employment, commercial activities, schooling, and personal security—for a span of nearly three decades.

Despite offers by the African Union (AU) and other neighbouring states to help mediate a dialogue in hopes of reaching a peaceful resolution,^42^ Prime Minister Abiy Ahmed has declined, arguing that it is an internal dispute, and as such, requires a domestic solution. This is disingenuous, as Abiy Ahmed has welcomed the involvement of Eritrea and Somalia,^43^ generating reports of war crimes by military agents of the former. Both sides in the conflict—the Tigray People’s Liberation Front (TPLF) and Ethiopian government forces—have been accused of war crimes amid military advances and setbacks. Armed drones from the Middle East and Turkey have helped the Ethiopian armed forces push the TPLF away from Addis Ababa and back to the Tigray region, while the TPLF was recently bolstered by its alliance with the Oromo Liberation Army. Although the Abiy Ahmed regime allowed humanitarian assistance to enter the country in 2021^44^—and a “humanitarian truce” announced in March 2022 (to permit humanitarian aid reach those in need) barely held and finally collapsed roughly six months later—overtures from the Intergovernmental Authority on Development (IGAD) and the “A3+1” mediation group (Kenya, Niger, Tunisia, and Saint Vincent and the Grenadines) have been largely rebuffed.

For scholars of regionalisms, the Tigray case is best described as an emerging case of bifurcated interregionalisms. The offers of formal regionalism bodies such as the AU and IGAD to mediate could readily transform into humanitarian interventions—especially if the expected malnutrition and starvation rates spike owing to increasing food prices (due to restricted Russian and disrupted Ukrainian exports of wheat and fertilizers—the latter serving as a key input for crops). These formal regionalisms will interact to a greater degree with extant informal regionalisms supported by militarized ethnic identities among state and non-state actors (e.g., NSAGs) alike in and around Tigray, including cross-border linkages with Eritrea and Sudan. This will generate an intense, illiberal regionness in this part of East Africa, akin to some of the regionness witnessed in the Horn of Africa, and moving away from the expected emancipatory outcomes envisioned by agential constructivism.

## Conclusions

There are many Africa*s*, and there are many bifurcated interregionalism*s*. As a relatively recent epistemological innovation within the new regionalisms literature, bifurcated interregionalism*s* have been applied to two African cases thus far—but could be readily employed to study regional dynamics in other parts of the continent (e.g., the DRC) and the globe. For instance, despite its particularly acrimonious relationship with East African Community (EAC) member-state Rwanda, the DRC became a full-fledged member of the regional bloc in July 2022—which now becomes an intriguing case of bifurcated interregionalisms. Despite being fellow members of the EAC regional intergovernmental organization, the DRC, Rwanda, and Uganda have government armed forces conducting small-scale cross-border operations against one another, support NSAGs (some of which are driven by militarization and ethnic-identity-building dynamics on each side of and crossing the border), and have state and non-state actors who benefit from networks of plunder across the region.^45^ By the same token, there are many extant and possible order*s* in the international system. Praxes of regionalism—that inculcate agential constructivism*s*—recognize that the uncertainty surrounding order in the international system casts formal regionalism as less attractive or lacking the ability to generate confidence among its residents in terms of the provision of public goods, ranging from equitable and sustainable development to personal security. Bifurcated interregionalisms are useful analytical lenses through which to understand these trends as well as their impact on global, regional, and local order*s*.

It is important to keep in mind that bifurcated interregionalisms, though important and illuminating as connective “sinews and tissues,” are but one facet of the current dynamics driving order in the international system. It is against this backdrop that this article poses the following question by way of concluding remarks: is (dis)order in the international system at the dawn of the 2020s any different from past decades? COVID-19 was an auspicious event that impacted all regions across the globe—with myriad development and human security ramifications.^46^ Yet, at the start of each decade, there seems to be an uptick of anxieties towards the coming decade’s emerging order. Reflecting on a tumultuous decade of the 1970s, Shaw expresses concern for ecological (read: environmental) limits of inequitable economic growth at the dawn of the 1980s as well as the rise of “new nationalism” and its impact on order in the international system. In a short monograph first presented at the International Political Science Association (IPSA) meetings in Moscow in mid 1979, and published by Dalhousie’s Centre for Foreign Policy Studies (the precursor to the Centre for the Study of Security and Development) a year later with comments by Robert O. Matthews (University of Toronto) and Robert McKinnell (Canadian International Development Agency), respectively, Shaw concludes that the international system at the start of the 1980s:

…is at an historic conjuncture in which inter-imperial rivalries, the rise of NICs [newly industrialized countries] and the appearance of ‘new nationalism’ together make anarchy a possible future scenario. Some variant of enlightened internationalism would seem to be an imperative to avoid such a fate and to lay the foundation for a more equitable and sustainable international political economy, one in which genuine interdependence is the popular motif.^47^

Enlightened internationalism did not make much headway in the 1980s as Soviet-US rivalries continued to dominate the international system, and scholars still debate the depth of human security advances hastened by the end of the Cold War and growth of international institutions in the 1990s.

At the dawn of the 2020s, however, we see similar conditions, with BRICSAM (Brazil, Russia, India, China, South Africa, the ASEAN states, and Mexico) replacing the NICs, *mutatis mutandis*. Increasingly dire warnings issued by epistemic communities concerning global warming—reflected in the concerns of Lucian Ashworth^48^—not to mention the lingering impact of COVID on local, regional, and global public health as well as global supply chains—are layered upon geopolitical sabre-rattling and new weapon systems tests by Russia and China. Pundits ruminate on whether the post-COVID era will generate a “back to the future” of either the rapid yet unequal economic growth of the “roaring 1920s” or the toxic mix of nationalism and extremism, driven by a contemporary take on populism, akin to the grievance-inducing “beggar-thy-neighbour 1930s.” Though extant regionalisms and the multitude of influential global “governors” and “actors” of the present decade make the international system qualitatively different from a century ago, Brexit has demonstrated the fragility of the formal regionalist project. Though anarchy as postulated by Shaw in 1980 is an unlikely outcome, the spectre of “new nationalism” he envisioned more than four decades ago—conceptualized in the plural and incorporating populisms for the 2020s (i.e., new nationalism*s*)—should not be dismissed by observers seeking to understand the contours of contemporary order in the international system. Indeed, we may be witnessing the creation of illiberal regionalisms animating Russia, China, and Turkey, among others, and incorporating non-state actors that thrive under such conditions. In a similar vein, the bifurcated interregionalisms in and around the Mozambican province of Cabo Delgado and Ethiopia’s Tigray region exhibit illiberal regionalisms via the interactions and contestations between state and non-state actors. Accounting for the establishment, contestation, and diffusion of illiberal norms would help refine agential constructivism, which has thus far tended to focus exclusively upon the emancipatory (read: liberal) potential of transnational norms and their state and non-state agents. As a result, the defining feature of the 2020s might be illiberal regionness—driven by the interactions and norm contestations of constituent actors seeking to secure illiberal regional objectives—thereby generating novel iterations of bifurcated interregionalisms.

## Notes

1 Including, but not limited to, fieldwork conducted in Sierra Leone in 2003, 2008, and 2010, Ghana in 2007, 2008, and 2016, Uganda in 2016, South Africa in 2003, 2013, and 2015, and the Democratic Republic of Congo in 2022.

2 See, for example, Timothy M. Shaw, “New regionalisms in Africa in the new millennium: Comparative perspectives on renaissance, realisms and/or regressions,” *New Political Economy* 5, no. 3 (2000): 399–414; Timothy M. Shaw, “African foreign policy in the new millennium: From coming anarchies to security communities? From new regionalisms to new realisms?” in Kevin C. Dunn and Timothy M. Shaw, eds., *Africa’s Challenge to International Relations Theory* (Basingstoke: Macmillan, 2001), 204–219; Timothy M. Shaw, “Which challenges for African regional agency? Prospects for the continent on the road to 2020,” in Ulrike Lorenz-Carl and Martin Rempe, eds., *Mapping Agency: Comparing Regionalisms in Africa* (Aldershot: Ashgate, 2013), 175–186; Timothy M. Shaw, J. Andrew Grant, and Scarlett Cornelissen, “Introduction and overview: The study of new regionalism(s) at the start of the second decade of the twenty-first century,” in Timothy M. Shaw, J. Andrew Grant, and Scarlett Cornelissen, eds., *The Ashgate Research Companion to Regionalisms* (Aldershot: Ashgate, 2012), 3–30; Timothy M. Shaw, Fredrik Söderbaum, Julius E. Nyang’oro, and J. Andrew Grant, “The future of new regionalism in Africa: Regional governance, human security/development and beyond,” in J. Andrew Grant and Fredrik Söderbaum, eds., *The New Regionalism in Africa* (Aldershot: Ashgate, 2003), 192–206.

3 W.R. Nadège Compaoré, Stéphanie Martel, and J. Andrew Grant, “Reflexive pluralism in IR: Canadian contributions to worlding the Global South,” *International Studies Perspectives* 23, no. 1 (2022): 71–93.

4 Hannah Arendt, *The Human Condition* (Chicago: University of Chicago Press, 1958).

5 Larry Swatuk, “Theorizing global political economy in an era of ferment,” paper presented at the SSHRC Connection Grant workshop, “Centres, peripheries, interstices: Towards an eclectic political economy of global development,” Ottawa, SecDev Foundation, 10–11 June 2022.

6 Ibid.

7 Björn Hettne and Fredrik Söderbaum, “The new regionalism approach,” *Politeia* 17, no. 3 (1998): 6–21; Björn Hettne and Fredrik Söderbaum, “Theorising the rise of regionness,” *New Political Economy* 5, no. 3 (2000): 457–473.

8 Hettne and Söderbaum, “Theorising the rise of regionness,” 461.

9 Ibid.

10 Emanuel Adler and Vincent Pouliot, “International practices,” *International Theory* 3, no. 1 (2011): 1–36; J. Andrew Grant, “Agential constructivism and change in world politics,” *International Studies Review* 20, no. 2 (2018): 255–263; J. Andrew Grant, Abdiasis Issa, and Badriyya Yusuf, “Agential constructivism, shadow regionalisms and interregional dynamics in the Horn of Africa,” in Elisa Lopez Lucia and Frank Mattheis, eds., *The Unintended Consequences of Interregionalism: Effects on Regional Actors, Societies and Structures* (London: Routledge, 2021), 165–184; J. Andrew Grant and Cindy Wilhelm, “A flash in the pan? Agential constructivist perspectives on local content, governance, and the large-scale mining–artisanal and small-scale mining interface in West Africa,” *Resources Policy* 77, no. 102592 (2022): 1–11.

11 Erin Baines, “Documenting the unspeakable: Relational methodologies, militarized masculinities, and paternal love,” paper presented at the SSHRC Connection Grant workshop, “Centres, peripheries, interstices: Towards an eclectic political economy of global development,” Halifax, Dalhousie University, 29–30 October 2021.

12 Jane Parpart, “Rethinking silence, voice and the in-between: Exploring a world in flux,” paper presented at the SSHRC Connection Grant workshop, “Centres, peripheries, interstices: Towards an eclectic political economy of global development,” Ottawa, SecDev Foundation, 10–11 June 2022.

13 Alexander Wendt, *Social Theory of International Politics* (Cambridge: Cambridge University Press, 1999).

14 Nicholas Onuf, *Making Sense, Making Worlds: Constructivism in Social Theory and International Relations* (London: Routledge, 2013).

15 Grant, “Agential constructivism and change in world politics.”

16 See, for example, Shaw, “African foreign policy in the new millennium”; contributors to Fredrik Söderbaum and Timothy M. Shaw, eds., *Theories of New Regionalism: A Palgrave Reader* (Basingstoke: Palgrave Macmillan, 2003); Shaw et al., “The future of new regionalism in Africa”; Morten Bøås, Marianne H. Marchand, and Timothy M. Shaw, “The weave-world: The regional interweaving of economies, ideas and identities,” in Söderbaum and Shaw, *Theories of New Regionalism*, 192–210; contributors to Shaw et al., *The Ashgate Research Companion to Regionalisms*; Shaw, “Which challenges for African regional agency?”; Abigail Kabandula and Timothy M. Shaw, “Rising powers and the Horn of Africa: Conflicting regionalisms,” *Third World Quarterly* 39, no. 12 (2018): 2315–2333.

17 See, for example, Okechukwu C. Iheduru, “New regionalism, states and non-state actors in West Africa,” in Grant and Söderbaum, *The New Regionalism in Africa*, 47–66; Okechukwu. C. Iheduru, “The ‘new’ ECOWAS: Implications for the study of regional integration,” in Shaw et al., *The Ashgate Research Companion to Regionalisms*, 213–239; Daniel C. Bach, “Revisiting a paradigm,” in Daniel C. Bach ed., *Regionalization in Africa: Integration and Disintegration* (Oxford: James Currey, 1999), 1–14; Daniel C. Bach, “New regionalism as an alias: Regionalization through trans-state networks,” in Grant and Söderbaum, *The New Regionalism in Africa*, 21–30; Sandra J. MacLean, “New regionalism and conflict in the Democratic Republic of Congo: Networks of plunder and networks for peace,” in Grant and Söderbaum, *The New Regionalism in Africa*, 110–124; Fredrik Söderbaum and Ian Taylor, “The role of the state in the Maputo Development Corridor,” in Fredrik Söderbaum and Ian Taylor eds., *Regionalism and Uneven Development: The Case of the Maputo Development Corridor* (Aldershot: Ashgate, 2003), 41–52; Fredrik Söderbaum and Ian Taylor, eds., *Micro-Regionalism in West Africa—Evidence from Two Case Studies*, Discussion Paper 34 (Uppsala: Nordiska Afrikainstitutet, 2007); contributors to Grant and Söderbaum, *The New Regionalism in Africa*; James J. Hentz and Morten Bøås, eds., *New and Critical Security and Regionalism: Beyond the Nation-State* (Aldershot: Ashgate, 2003); Fredrik Söderbaum, *The Political Economy of Regionalism: The Case of Southern Africa* (Basingstoke: Palgrave Macmillan, 2004); Fredrik Söderbaum, “Formal and informal regionalism,” in Shaw et al., *The Ashgate Research Companion to Regionalisms*, 51–67; Fredrik Söderbaum, *Rethinking Regionalism* (London: Palgrave Macmillan, 2016); contributors to Fredrik Söderbaum and Ian Taylor, eds., *Afro-Regions: The Dynamics of Cross-Border Micro-Regionalism in Africa* (Uppsala: Nordic Africa Institute, 2008); J. Andrew Grant and Thomas Kwasi Tieku, “New regionalisms and the African Union: Reflections on the rise of Africrats, regional economic integration, and inter-regional relations,” in B.J.C. McKercher, ed., *Routledge Handbook of Diplomacy and Statecraft* (London: Routledge, 2012), 264–273; J. Andrew Grant, Matthew I. Mitchell, Frank K. Nyame, and Natalia Yakovleva, “Micro-regionalisms, information and communication technologies, and migration in West Africa: A comparative analysis of Ghana’s diamond, cocoa, and gold sectors,” in Lorenz-Carl and Rempe, *Mapping Agency*, 149–174; J. Andrew Grant, Abdiasis Issa, Fredrik Söderbaum, and Badriyya Yusuf, “Regionalism beyond state-centrism: African regionalism in comparative perspective,” paper presented at the SSHRC Connection Grant workshop, “Centres, peripheries, interstices: Towards an eclectic political economy of global development,” Ottawa, SecDev Foundation, 10–11 June 2022; contributors to Lorenz-Carl and Rempe, *Mapping Agency*; contributors to Ulf Engel, Heidrun Zinecker, Frank Mattheis, Antje Dietze, and Thomas Plötze, eds., *The New Politics of Regionalism: Perspectives from Africa, Latin America and Asia-Pacific* (London: Routledge, 2017); Frank Mattheis, *New Regionalism in the South: Mercosur and SADC in a Comparative and Interregional Perspective* (Leipzig: Leipziger Universitätsverlag, 2014); Frank Mattheis, Luca Raineri, and Alessandra Russo, *Fringe Regionalism: When Peripheries Become Regions* (London: Palgrave, 2019); Grant et al., “Agential constructivism, shadow regionalisms.”

18 Raimo Väyrynen, “Regionalism: Old and new,” *International Studies Review* 5, no. 1 (2003): 25–51; Andrew Hurrell, “One world? Many worlds? The place of regions in the study of international society,” *International Affairs* 83, no. 1 (2007): 127–146; Sonia Le Gouriellec, “Regional power and contested hierarchy: Ethiopia, an ‘imperfect hegemon’ in the Horn of Africa,” *International Affairs* 94, no. 5 (2018): 1059–1075; Elisa Lopez Lucia, “Rethinking regionalism and the politics of regionalisation: The performance of ECOWAS’s agency by Nigeria and the European Union,” *Journal of International Relations and Development* 21, no. 3 (2018): 663–688.

19 See, for example, the contributors to Lopez Lucia and Mattheis, *The Unintended Consequences of Interregionalism*.

20 Frank Mattheis, “Towards bifurcated regionalism: The production of regional overlaps in Central Africa,” in Engel et al., *The New Politics of Regionalism*, 57–71; Lopez Lucia and Mattheis, *Unintended Consequences of Interregionalism*, 2021; Mattheis et al., *Fringe Regionalism*; Grant et al., “Agential constructivism, shadow regionalisms.”

21 Heiner Hänggi, Ralf Roloff, and Jürgen Rüland, “Interregionalism: A new phenomenon in international relations,” in Heiner Hänggi, Ralf Roloff, and Jürgen Rüland, eds., *Interregionalism and International Relations* (London: Routledge, 2006), 3–14; Ralf Roloff, “Interregionalism in theoretical perspective: State of the art,” in Hänggi et al., *Interregionalism and International Relations*, 17–30; Grant et al., “Agential constructivism, shadow regionalisms.”

22 Mary Farrell, “A move toward hybrid interregionalism in Asia,” in Fredrik Söderbaum and Patrik Stålgren, eds., *The European Union and the Global South* (Boulder: Lynne Rienner, 2010), 115–140; Bart Gaens, “The rise of interregionalisms: The case of the European Union’s relations with East Asia,” in Shaw et al., *The Ashgate Research Companion to Regionalisms*, 69–87; Björn Hettne and Frederik Ponjaert, “Interregionalism and world order: The diverging EU and US models,” in Mario Telò, ed., *European Union and New Regionalism: Competing Regionalism and Global Governance in a Post-Hegemonic Era*, 2nd Edition (London: Routledge, 2014), 115–138.

23 Gian Luca Gardini and Andrés Malamud, “Debunking interregionalism: Concepts, types and critique—with a pan-Atlantic focus,” *Interregionalism Across the Atlantic Space: United Nations University Series on Regionalism, Volume 15* (London: Springer, 2018), 15–31.

24 Grant et al., “Agential constructivism, shadow regionalisms,” 166–167.

25 Jessica Trisko Darden and Emily Estelle, *Confronting Islamist Insurgencies in Africa: The Case of the Islamic State in Mozambique* (Foreign Policy Research Institute, Summer 2021), 432.

26 Francisco Almeida dos Santos, “War in resource-rich Northern Mozambique—Six scenarios,” CHR Michelsen Institute Insight Report 02, 2020; ICG, “Stemming the insurrection in Mozambique’s Cabo Delgado,” International Crisis Group, Africa Report no. 303, 2021; Trisko Darden and Estelle, *Confronting Islamist Insurgencies in Africa*; Makaita Noel Mutasa and Cyprian Muchemwa, “Ansar Al-Sunna Mozambique: Is it the Boko Haram of Southern Africa?” *Journal of Applied Security Research* 17, no. 8 (2021): 332–351.

27 ICG, “Stemming the insurrection in Mozambique’s Cabo Delgado.”

28 Cited in Trisko Darden and Estelle, *Confronting Islamist Insurgencies in Africa*.

29 MacLean, “New regionalism and conflict in the Democratic Republic of Congo”; J. Andrew Grant, W.R. Nadège Compaoré, Matthew I. Mitchell, and Timothy M. Shaw, “Prospects and trends in the governance of Africa’s natural resources: Reflections on the role of external and internal actors,” in J. Andrew Grant, W.R. Nadège Compaoré, and Matthew I. Mitchell, eds., *New Approaches to the Governance of Natural Resources: Insights from Africa* (London: Palgrave Macmillan, 2014), 267–284; J. Andrew Grant, “Human security and the G-8 Africa Action Plan: What provisions for women and children in post-conflict communities?” in Rosalind Boyd, ed., *The Search for Lasting Peace: Critical Perspectives on Gender-Responsive Human Security* (Aldershot: Ashgate, 2014), 151–167.

30 ICG, “Stemming the insurrection in Mozambique’s Cabo Delgado.”

31 MacLean, “New regionalism and conflict in the Democratic Republic of Congo”; J. Andrew Grant, “Informal cross-border micro-regionalism in West Africa: The case of the Parrot’s Beak,” in Söderbaum and Taylor, *Afro-Regions*, 105–120.

32 IPSS, *Mozambique Conflict Insight Peace & Security Report*, Institute for Peace and Security Studies, vol. 1, 2020.

33 Ibid.; IISS Strategic Comments, “The Islamist insurgency in Mozambique,” The International Institute for Strategic Studies, vol. 27, Comment 22, 2021, 59–72.

34 Bulama Bukarti and Sandun Munasinghe, *The Mozambique Conflict and Deteriorating Security Situation* (London: The Tony Blair Institute for Global Change, 2020); Eric Morier-Genoud, “The Jihadi insurgency in Mozambique: Origins, nature and beginning,” *Journal of Eastern African Studies* 14, no. 3 (2020): 396–412; Samuel Kehinde Okunade, Olumuyiwa Temitope Faluyi, and Emmanuel Matambo, “Evolving patterns of insurgency in Southern and West Africa: Refocusing the Boko Haram lens on Mozambique,” *African Security Review* 30, no. 4 (2021): 434–450; Trisko Darden and Estelle, *Confronting Islamist Insurgencies in Africa*; Paulo Conceição João Faria, “The rise and root causes of Islamic insurgency in Mozambique and its security implication to the region,” The Institute for Peace and Security Studies, Policy Brief vol. 15, 2021.

35 Felix Makonye, “The Cabo Delgado insurgency in Mozambique: Origin, ideology, recruitment strategies and social, political and economic implications for natural gas and oil exploration,” *African Journal of Terrorism and Insurgency Research* 1, no. 3 (2020): 59–72; Almeida dos Santos, “War in resource-rich Northern Mozambique.”

36 Charis Enns, Nathan Andrews, and J. Andrew Grant, “Security for whom? Analysing hybrid security governance in Africa’s extractive sectors,” *International Affairs* 96, no. 4 (2020): 995–1013.

37 Mekonen Aregai and Muluberhan Bedemariam, “Socio-environmental conflicts between the refugee populations and their host communities: The case of Eritrean refugees in North Western Tigray, Ethiopia,” *Environmental & Socio-Economic Studies* 8, no. 2 (2020): 54–62. ARBNS, “Ethiopia: Never-ending Tigray conflict violent unrest extends far beyond the troubled north,” Africa Research Bulletin National Security 23128, 2021; Romina Istratii, “War and domestic violence: A rapid scoping of the international literature to understand the relationship and to inform current responses in the Tigray humanitarian crisis,” Working Paper 2, in *Bridging Religious Studies & Gender, Development and Public Health to Address Domestic Violence in Religious Communities*, SOAS University of London, 2021; Eritrea Focus and Oslo Analytica, *The Tigray War and Regional Implications* (Eritrea Focus and Oslo Analytica, Vol. 1, 2021).

38 Dedefo Bedaso, *Human Rights Crisis in Tigray Region of Ethiopia: The Extent of International Intervention and PM Abiy Ahmed’s Denial of Humanitarian Access into the Region* (Cincinnati: University of Cincinnati School of Law, 2020–2021).

39 See, for example, Philippe Pellet, *Understanding the 2020–2021 Tigray Conflict in Ethiopia—Background, Root Causes, and Consequences*, KKI Institute for Foreign Affairs and Trade Policy Brief KE-2021/39, 2021.

40 Bedaso, *Human Rights Crisis in Tigray*, 2.

41 OCHR, *Ethiopia – Northern Ethiopia Humanitarian Update Situation Report* (Office of the United Nations High Commissioner for Human Rights, 2021); Aleksi Ylönen, “Counting on friends in Tigray: Internal and regional considerations in the ongoing crisis,” *The Horn Bulletin* IV, no. II (2021).

42 Anne-Eleonore Deleersynder, *Ethiopia’s Tigray Conflict: Exposing the Limits of EU and AU Early Warning Mechanisms* (Brussels: The Multinational Development Policy Dialogue, KAS, 2021); Ylönen, “Counting on friends in Tigray.”

43 Ylönen, “Counting on friends in Tigray.”

44 Zerihun A. Woldeselassieessure, *Why International Pressure is Not Helping End Violence in Ethiopia* (International Peace Institute Global Observatory, 2021).

45 Raynold Wonder Alorse, W.R. Nadège Compaoré, and J. Andrew Grant, “Assessing the European Union’s engagement with transnational policy networks on conflict-prone natural resources,” *Contemporary Politics* 21, no. 3 (2015): 245–257; MacLean, “New regionalism and conflict in the Democratic Republic of Congo”; J. Andrew Grant, “Eliminating conflict diamonds and other conflict-prone minerals,” in Katharina P. Coleman and Thomas K. Tieku, eds., *African Actors in International Security: Shaping Contemporary Norms* (Boulder: Lynne Rienner, 2018), 51–71; J. Andrew Grant, “Conflict-prone minerals, forced migration and norm dynamics in the Kimberley Process and ICGLR,” in Tim Krieger, Diana Panke, and Michael Pregernig, eds., *Environmental Conflicts, Migration and Governance* (Bristol: Bristol University Press, 2020), 197–217; Wilder Alejandro Sanchez, “DRC joins the East African Community,” *Geopolitical Monitor*, 20 July 2022.

46 Timothy M. Shaw, “‘Global governance and human security’ after 2021: Beyond COVID-19 and global warming,” paper presented at the SSHRC Connection Grant workshop, “Centres, peripheries, interstices: Towards an eclectic political economy of global development,” Ottawa, SecDev Foundation, 10–11 June 2022.

47 Timothy M. Shaw, *Towards an International Political Economy for the 1980s: From Dependence to (Inter)dependence* (Halifax: Centre for Foreign Policy Studies, Dalhousie University, 1980): 58. For related reflections that focus on Africa’s role in the international system at the dawn of the 1980s, see Timothy M. Shaw, “Foreign policy, political economy and the future: Reflections on Africa in the world system,” *African Affairs* 79, no. 315 (1980): 260–268; Timothy M. Shaw, “From dependence to self-reliance: Africa’s prospects for the next twenty years,” *International Journal* 35, no. 4 (1980): 821–844.

48 Lucian Ashworth, “Global governance in the Anthropocenes,” paper presented at the SSHRC Connection Grant workshop, “Centres, peripheries, interstices: Towards an eclectic political economy of global development,” Ottawa, SecDev Foundation, 10–11 June 2022.

